# 2,2-Dichloro-*N*-(4-chloro­phenyl­sulfon­yl)­acetamide

**DOI:** 10.1107/S1600536808021715

**Published:** 2008-07-19

**Authors:** B. Thimme Gowda, Sabine Foro, P. G. Nirmala, B. P. Sowmya, Hartmut Fuess

**Affiliations:** a Department of Chemistry, Mangalore University, Mangalagangotri 574 199, Mangalore, India; b Institute of Materials Science, Darmstadt University of Technology, Petersenstrasse 23, D-64287 Darmstadt, Germany

## Abstract

In the crystal structure of the title compound (N4CPSDCAA), C_8_H_6_Cl_3_NO_3_S, the conformations of the N—H and C=O bonds in the SO_2_—NH—CO—C group are *trans* to each other, similar to those observed in 2,2-dichloro-*N*-(phenyl­sulfon­yl)­acetamide (NPSDCAA), 2,2-dichloro-*N*-(4-methyl­phenyl­sulfon­yl)acetamide (N4MPSDCAA) and *N*-(4-chloro­phenyl­sulfon­yl)-2,2,2-trimethyl­acetamide (N4CPSTMAA), with similar bond parameters. The –SNHCOC– unit in N4CPSDCAA is essentially planar and makes a dihedral angle of 79.67 (5)° with the benzene ring, comparable to 79.75 (8)° in NPSDCAA, 81.02 (5)° in N4MPSDCAA and 82.2 (1)° in N4CPSTMAA. The mol­ecules in N4CPSDCAA are linked into layers parallel to the (001) plane by inter­molecular N—H⋯O hydrogen bonds.

## Related literature

For related literature, see: Gowda *et al.* (2003 ▶, 2006 ▶); Gowda, Foro, Nirmala *et al.* (2008 ▶); Gowda, Foro, Sowmya *et al.* (2008 ▶).
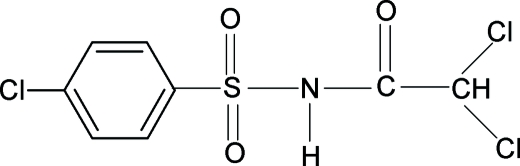


## Experimental

### 

#### Crystal data

C_8_H_6_Cl_3_NO_3_S
*M* *_r_* = 302.55
Orthorhombic, 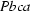

*a* = 9.5909 (5) Å
*b* = 10.1750 (5) Å
*c* = 23.256 (1) Å
*V* = 2269.49 (19) Å^3^
*Z* = 8
Mo *K*α radiation
μ = 0.98 mm^−1^
*T* = 299 (2) K
0.32 × 0.28 × 0.08 mm

#### Data collection

Oxford Diffraction Xcalibur diffractometer
Absorption correction: multi-scan (*CrysAlis RED*; Oxford Diffraction, 2007 ▶) *T*_min_ = 0.745, *T*_max_ = 0.926
10378 measured reflections
2309 independent reflections
1642 reflections with *I* > 2σ(*I*)
*R*_int_ = 0.020

#### Refinement

*R*[*F* ^2^ > 2σ(*F* ^2^)] = 0.027
*wR*(*F* ^2^) = 0.073
*S* = 1.08
2309 reflections
145 parameters
H-atom parameters constrained
Δρ_max_ = 0.34 e Å^−3^
Δρ_min_ = −0.27 e Å^−3^

### 

Data collection: *CrysAlis CCD* (Oxford Diffraction, 2004 ▶); cell refinement: *CrysAlis RED* (Oxford Diffraction, 2007 ▶); data reduction: *CrysAlis RED*; program(s) used to solve structure: *SHELXS97* (Sheldrick, 2008 ▶); program(s) used to refine structure: *SHELXL97* (Sheldrick, 2008 ▶); molecular graphics: *PLATON* (Spek, 2003 ▶); software used to prepare material for publication: *SHELXL97*.

## Supplementary Material

Crystal structure: contains datablocks I, global. DOI: 10.1107/S1600536808021715/ci2630sup1.cif

Structure factors: contains datablocks I. DOI: 10.1107/S1600536808021715/ci2630Isup2.hkl

Additional supplementary materials:  crystallographic information; 3D view; checkCIF report

## Figures and Tables

**Table 1:** Hydrogen-bond geometry (Å, °)

*D*—H⋯*A*	*D*—H	H⋯*A*	*D*⋯*A*	*D*—H⋯*A*
N1—H1N⋯O3^i^	0.86	1.97	2.814 (2)	169

Symmetry code: (i) 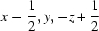.

**Fig. 1.**
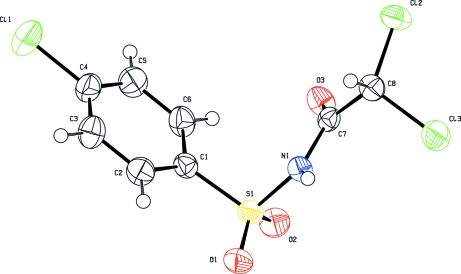
Molecular structure of the title compound, showing the atom labeling scheme. Displacement ellipsoids are drawn at the 50% probability level. H atoms are represented as small spheres of arbitrary radii.

**Fig. 2.**
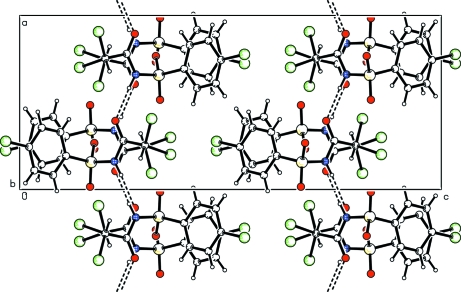
Molecular packing of the title compound with hydrogen bonding shown as dashed lines.

## supplementary crystallographic information

### Comment

As part of a study of the substituent effects on the crystal structures of *N*-(aryl)-sulfonamides and substituted amides, in the present work, the structure of *N*-(4-chlorophenylsulfonyl)-2,2-dichloroacetamide (N4CPSDCAA) has been determined (Gowda *et al.*, 2003, 2006; Gowda, Foro, Nirmala *et al.*, 2008; Gowda, Foro, Sowmya *et al.*, 2008). The conformations of the N—H and C═O bonds of the SO_2_—NH—CO—C group in N4CPSDCAA are *trans* to each other (Fig. 1), similar to those observed in *N*-(phenylsulfonyl)-2,2-dichloroacetamide (NPSDCAA), *N*-(4-methylphenylsulfonyl)-2,2-dichloroacetamide (N4MPSDCAA) (Gowda, Foro, Nirmala *et al.*, 2008) and (4-chlorophenylsulfonyl)-2,2,2- trimethylacetamide (N4CPSTMAA) (Gowda, Foro, Sowmya *et al.*, 2008). The bond parameters in N4CPSDCAA are similar to those in NPSDCAA, N4MPSDCAA, N4CPSTMAA (Gowda, Foro, Nirmala *et al.*, 2008; Gowda, Foro, Sowmya *et al.*, 2008), *N*-(aryl)-2,2-dichloro- acetamides (Gowda *et al.*, 2006) and 4-chlorobenzenesulfonamide (Gowda *et al.*, 2003).

The packing diagram of N4CPSDCAA showing the N—H···O hydrogen bonds (Table 1) involved in the formation of layers parallel to the (0 0 1) plane is shown in Fig. 2.

### Experimental

The title compound was prepared by refluxing 4-chlorobenzenesulfonamide (0.10 mole) with excess dichloroacetyl chloride (0.20 mole) for about an hour on a water bath. The reaction mixture was cooled and poured into ice cold water. The resulting solid was separated, washed thoroughly with water and dissolved in warm dilute sodium hydrogen carbonate solution. The title compound was precipitated by acidifying the filtered solution with glacial acetic acid. It was filtered, dried and recrystallized from ethanol. The purity of the compound was checked by determining its melting point. It was characterized by recording its infrared and NMR spectra. Single crystals of the title compound used for X-ray diffraction studies were obtained by slow evaporation of an ethanolic solution.

### Refinement

H atoms were positioned with idealized geometry (C-H = 0.93 or 0.98 Å, N-H = 0.86 Å) and were refined using a riding model, with U_iso_(H) = 1.2*U*_eq_(C,N). To improve R1, wR2 and S values (1 0 2) reflection was omitted during the refinement

### Crystal data

**Table:**

C_8_H_6_Cl_3_NO_3_S	*F*_000_ = 1216
*M**_r_* = 302.55	*D*_x_ = 1.771 Mg m^−^^3^
Orthorhombic, *P**b**c**a*	Mo *K*α radiation λ = 0.71073 Å
Hall symbol: -P 2ac 2ab	Cell parameters from 5145 reflections
*a* = 9.5909 (5) Å	θ = 2.2–28.0º
*b* = 10.1750 (5) Å	µ = 0.98 mm^−^^1^
*c* = 23.256 (1) Å	*T* = 299 (2) K
*V* = 2269.49 (19) Å^3^	Plate, colourless
*Z* = 8	0.32 × 0.28 × 0.08 mm

### Data collection

**Table:**

Oxford Diffraction Xcalibur diffractometer	2309 independent reflections
Radiation source: fine-focus sealed tube	1642 reflections with *I* > 2σ(*I*)
Monochromator: graphite	*R*_int_ = 0.020
*T* = 299(2) K	θ_max_ = 26.4º
ω and φ scans	θ_min_ = 3.1º
Absorption correction: multi-scan(CrysAlis RED; Oxford Diffraction, 2007)	*h* = −11→11
*T*_min_ = 0.745, *T*_max_ = 0.926	*k* = −10→12
10378 measured reflections	*l* = −28→29

### Refinement

**Table:**

Refinement on *F*^2^	Secondary atom site location: difference Fourier map
Least-squares matrix: full	Hydrogen site location: inferred from neighbouring sites
*R*[*F*^2^ > 2σ(*F*^2^)] = 0.027	H-atom parameters constrained
*wR*(*F*^2^) = 0.073	*w* = 1/[σ^2^(*F*_o_^2^) + (0.0279*P*)^2^ + 1.2816*P*] where *P* = (*F*_o_^2^ + 2*F*_c_^2^)/3
*S* = 1.09	(Δ/σ)_max_ = 0.001
2309 reflections	Δρ_max_ = 0.34 e Å^−^^3^
145 parameters	Δρ_min_ = −0.27 e Å^−^^3^
Primary atom site location: structure-invariant direct methods	Extinction correction: none

### Special details

**Table:**

Geometry. All e.s.d.'s (except the e.s.d. in the dihedral angle between two l.s. planes) are estimated using the full covariance matrix. The cell e.s.d.'s are taken into account individually in the estimation of e.s.d.'s in distances, angles and torsion angles; correlations between e.s.d.'s in cell parameters are only used when they are defined by crystal symmetry. An approximate (isotropic) treatment of cell e.s.d.'s is used for estimating e.s.d.'s involving l.s. planes.
Refinement. Refinement of *F*^2^ against ALL reflections. The weighted *R*-factor *wR* and goodness of fit *S* are based on *F*^2^, conventional *R*-factors *R* are based on *F*, with *F* set to zero for negative *F*^2^. The threshold expression of *F*^2^ > σ(*F*^2^) is used only for calculating *R*-factors(gt) *etc*. and is not relevant to the choice of reflections for refinement. *R*-factors based on *F*^2^ are statistically about twice as large as those based on *F*, and *R*- factors based on ALL data will be even larger.

### Fractional atomic coordinates and isotropic or equivalent isotropic displacement parameters (Å^2^)

**Table:**

	*x*	*y*	*z*	*U*_iso_*/*U*_eq_	
C1	0.1931 (2)	0.2061 (2)	0.10986 (9)	0.0312 (5)	
C2	0.0826 (2)	0.2583 (2)	0.07911 (10)	0.0397 (6)	
H2	−0.0087	0.2353	0.0882	0.048*	
C3	0.1095 (3)	0.3449 (3)	0.03482 (11)	0.0464 (6)	
H3	0.0364	0.3802	0.0136	0.056*	
C4	0.2445 (3)	0.3787 (2)	0.02219 (9)	0.0431 (6)	
C5	0.3549 (3)	0.3264 (3)	0.05226 (10)	0.0471 (6)	
H5	0.4458	0.3499	0.0430	0.056*	
C6	0.3295 (2)	0.2384 (2)	0.09643 (10)	0.0405 (6)	
H6	0.4031	0.2015	0.1168	0.049*	
C7	0.2795 (2)	0.2659 (2)	0.24629 (9)	0.0275 (5)	
C8	0.2532 (2)	0.3590 (2)	0.29700 (9)	0.0332 (5)	
H8	0.1789	0.4210	0.2869	0.040*	
N1	0.16103 (17)	0.21068 (16)	0.22462 (7)	0.0283 (4)	
H1N	0.0830	0.2314	0.2404	0.034*	
O1	0.01558 (16)	0.06430 (16)	0.16706 (7)	0.0436 (4)	
O2	0.26697 (17)	0.01306 (15)	0.17698 (7)	0.0424 (4)	
O3	0.39439 (14)	0.24435 (16)	0.22705 (6)	0.0379 (4)	
Cl1	0.27646 (9)	0.48942 (8)	−0.03310 (3)	0.0686 (2)	
Cl2	0.40650 (7)	0.44634 (6)	0.31294 (3)	0.04543 (17)	
Cl3	0.20101 (7)	0.26485 (7)	0.35760 (2)	0.04963 (19)	
S1	0.15710 (6)	0.10591 (5)	0.16960 (2)	0.03174 (15)	

### Atomic displacement parameters (Å^2^)

**Table:**

	*U*^11^	*U*^22^	*U*^33^	*U*^12^	*U*^13^	*U*^23^
C1	0.0315 (12)	0.0341 (12)	0.0280 (11)	−0.0011 (9)	0.0005 (9)	−0.0033 (9)
C2	0.0314 (12)	0.0452 (14)	0.0425 (13)	−0.0007 (11)	−0.0017 (10)	0.0020 (11)
C3	0.0477 (15)	0.0485 (15)	0.0431 (14)	0.0070 (12)	−0.0074 (12)	0.0085 (12)
C4	0.0581 (16)	0.0403 (14)	0.0310 (12)	−0.0046 (12)	0.0046 (12)	0.0020 (11)
C5	0.0397 (14)	0.0629 (17)	0.0386 (13)	−0.0102 (13)	0.0071 (11)	−0.0001 (12)
C6	0.0312 (13)	0.0557 (15)	0.0346 (12)	−0.0004 (11)	−0.0008 (10)	0.0001 (11)
C7	0.0255 (12)	0.0289 (11)	0.0280 (10)	0.0023 (9)	−0.0023 (9)	0.0051 (9)
C8	0.0322 (12)	0.0322 (12)	0.0352 (11)	0.0030 (9)	−0.0028 (10)	−0.0014 (10)
N1	0.0200 (9)	0.0353 (10)	0.0296 (9)	0.0004 (7)	0.0020 (7)	−0.0014 (8)
O1	0.0366 (9)	0.0498 (10)	0.0443 (9)	−0.0153 (8)	−0.0038 (7)	0.0004 (8)
O2	0.0464 (10)	0.0329 (9)	0.0481 (10)	0.0080 (8)	−0.0005 (8)	−0.0007 (7)
O3	0.0220 (8)	0.0523 (10)	0.0394 (9)	0.0023 (7)	−0.0003 (7)	−0.0074 (8)
Cl1	0.0915 (6)	0.0667 (5)	0.0475 (4)	−0.0120 (4)	0.0065 (4)	0.0189 (4)
Cl2	0.0487 (4)	0.0371 (3)	0.0505 (4)	−0.0099 (3)	−0.0066 (3)	−0.0048 (3)
Cl3	0.0497 (4)	0.0641 (4)	0.0350 (3)	−0.0146 (3)	0.0070 (3)	−0.0037 (3)
S1	0.0303 (3)	0.0313 (3)	0.0337 (3)	−0.0030 (2)	−0.0011 (2)	−0.0007 (2)

### Geometric parameters (Å, °)

**Table:**

C1—C6	1.385 (3)	C6—H6	0.93
C1—C2	1.385 (3)	C7—O3	1.209 (2)
C1—S1	1.757 (2)	C7—N1	1.364 (3)
C2—C3	1.380 (3)	C7—C8	1.534 (3)
C2—H2	0.93	C8—Cl2	1.758 (2)
C3—C4	1.371 (4)	C8—Cl3	1.776 (2)
C3—H3	0.93	C8—H8	0.98
C4—C5	1.376 (4)	N1—S1	1.6659 (17)
C4—Cl1	1.737 (2)	N1—H1N	0.86
C5—C6	1.384 (3)	O1—S1	1.4230 (16)
C5—H5	0.93	O2—S1	1.4257 (16)
			
C6—C1—C2	121.0 (2)	O3—C7—N1	123.20 (19)
C6—C1—S1	120.11 (17)	O3—C7—C8	123.12 (19)
C2—C1—S1	118.73 (17)	N1—C7—C8	113.68 (17)
C3—C2—C1	119.2 (2)	C7—C8—Cl2	109.69 (15)
C3—C2—H2	120.4	C7—C8—Cl3	108.86 (15)
C1—C2—H2	120.4	Cl2—C8—Cl3	109.94 (12)
C4—C3—C2	119.8 (2)	C7—C8—H8	109.4
C4—C3—H3	120.1	Cl2—C8—H8	109.4
C2—C3—H3	120.1	Cl3—C8—H8	109.4
C3—C4—C5	121.3 (2)	C7—N1—S1	124.49 (15)
C3—C4—Cl1	119.2 (2)	C7—N1—H1N	117.8
C5—C4—Cl1	119.4 (2)	S1—N1—H1N	117.8
C4—C5—C6	119.5 (2)	O1—S1—O2	120.86 (10)
C4—C5—H5	120.3	O1—S1—N1	104.11 (9)
C6—C5—H5	120.3	O2—S1—N1	108.35 (9)
C5—C6—C1	119.2 (2)	O1—S1—C1	109.09 (10)
C5—C6—H6	120.4	O2—S1—C1	109.54 (10)
C1—C6—H6	120.4	N1—S1—C1	103.40 (9)
			
C6—C1—C2—C3	−0.7 (3)	N1—C7—C8—Cl3	70.2 (2)
S1—C1—C2—C3	175.18 (18)	O3—C7—N1—S1	0.5 (3)
C1—C2—C3—C4	−0.5 (4)	C8—C7—N1—S1	179.96 (14)
C2—C3—C4—C5	1.1 (4)	C7—N1—S1—O1	173.55 (16)
C2—C3—C4—Cl1	−179.21 (19)	C7—N1—S1—O2	43.71 (19)
C3—C4—C5—C6	−0.4 (4)	C7—N1—S1—C1	−72.47 (18)
Cl1—C4—C5—C6	179.85 (19)	C6—C1—S1—O1	−167.61 (18)
C4—C5—C6—C1	−0.7 (4)	C2—C1—S1—O1	16.5 (2)
C2—C1—C6—C5	1.3 (3)	C6—C1—S1—O2	−33.3 (2)
S1—C1—C6—C5	−174.49 (18)	C2—C1—S1—O2	150.84 (17)
O3—C7—C8—Cl2	10.0 (3)	C6—C1—S1—N1	82.0 (2)
N1—C7—C8—Cl2	−169.47 (14)	C2—C1—S1—N1	−93.83 (19)
O3—C7—C8—Cl3	−110.4 (2)		

### Hydrogen-bond geometry (Å, °)

**Table:**

*D*—H···*A*	*D*—H	H···*A*	*D*···*A*	*D*—H···*A*
N1—H1N···O3^i^	0.86	1.97	2.814 (2)	169

Symmetry codes: (i) *x*−1/2, *y*, −*z*+1/2.
